# Developing occupational therapy and physiotherapy clinical support workers in their role in supporting student education

**DOI:** 10.1177/03080226251379996

**Published:** 2025-10-27

**Authors:** Gemma Bradley, Nina Bedding, Sarah Smith

**Affiliations:** 1 Faculty of Health and Wellbeing, Northumbria University, Newcastle Upon Tyne, UK; 2 Faculty of Health Sciences and Wellbeing, University of Sunderland, Sunderland, UK; 3 Institute of Health, University of Cumbria, Carlisle, UK

**Keywords:** Practice education, allied health professions, clinical support workers, role-development

## Abstract

**Introduction:**

Global healthcare services face increasing pressures and workforce shortages. But at the same time, there are increasing learners in practice as part of longer-term workforce strategies, which, in turn, create extra demands on educators and teams. Anecdotally, we understand that clinical support workers (CSWs) are sharing responsibilities for student learning, yet there is limited understanding of this involvement or activities to support role development. The aims of this study were to (1) explore the CSW role in the practice education of students and (2) evaluate a training programme for CSWs.

**Method:**

A mixed-methods design was utilised; 17 CSWs completed a survey prior to completing a training programme, and 11 participated in focus groups after the programme.

**Findings:**

We identified five themes: (1) *supporting students is an expectation*, (2) *supporting students ‘boosts the role’*, (3) *legitimacy of the CSW role to support students*, (4) *application of new learning to practice* and (5) *enablers and barriers to engagement with the programme*.

**Conclusion:**

CSWs are part of practice education ‘Communities of Practice’, evidenced through regular involvement with student learning. Responsibilities for student learning are perceived as adding value, although there is an absence of structures to legitimise the role. CSWs identified barriers and enablers to engaging with the programme, such as blended learning approaches and management support.

## Introduction and literature review

The Health and Care Professions Council (HCPC), who regulate the professions of occupational therapy (OT) and physiotherapy (PT), states that practice-based learning must be integral to all approved pre-registration education programmes for allied health professionals (AHPs) in the United Kingdom (Health and Care Professions Council (HCPC), 2017). However, the capacity for practice-based learning is under pressure. The National Health Service (NHS) Long Term Workforce Plan (NHS England, 2023) sets out priorities to increase numbers of AHP’s by 25% by 2031/2032 and to diversify opportunities for AHPs in practice (NHS England, 2023). One strategy to facilitate this is an increased number of pre-registration training places, which has resulted in more learners in practice.

At the same time as increasing numbers of learners, there are widely reported health and care workforce shortages (World Health Organization (WHO), 2022) with recent UK figures highlighting 112,000 vacancies in the NHS (NHS England, 2023). The number of registrants to facilitate and supervise practice education of students is struggling to keep pace with the number of learners. Alongside this, demand for healthcare services internationally continues to increase, exacerbated by the COVID-19 pandemic. As increasing demand and workforce shortages are global issues (WHO, 2022), such challenges are likely to also be of international concern.

In 2022, there were 379,133 clinical support staff in the NHS, made up of staff supporting a range of disciplines including doctors, nurses, midwives and AHPs (Nuffield Trust, 2022). Roles and titles can vary greatly between countries and across professions, with common job titles including clinical support worker (CSW), health care assistant, technical instructor and associate practitioner. Some roles are aligned to one specific discipline and some are aligned to clinical specialisms. Specific to AHPs, a recent scoping review highlighted wide variation in deployment and utilisation of the AHP support workforce internationally, dependent on many contextual factors such as discipline, setting and patient populations, the supervising registered practitioner and team structures (Etty et al., 2024).

Literature suggests some benefits of CSWs in terms of improved patient outcomes, increased patient satisfaction, increased intensity of clinical care alongside improved interdisciplinary working (Etty et al., 2024; Lizarondo et al., 2010). However, evidence of clinical and cost-effectiveness of delegating to AHP CSWs is limited to a small number of studies (Sarigiovannis et al., 2022). Sarigiovannis et al. also recognise many barriers to effective delegation such as limited training on delegation and supervision for registered staff, lack of clarity about roles and accountability, and negative perceptions or protectionism around roles.

Whilst numbers have increased and roles have diversified, this has happened largely without regulation, role boundaries or systematic education and training (Lewis and Kelly, 2015). Varied CSW roles reflect changing service needs but can lead to difficulties in identifying training opportunities and structured career progression and most CSWs report that training is informal, unstructured and ‘on-the-job’ (Ellis and Connell, 2001). The Australian Allied Health Assistant Framework (Queensland Government, 2022) and the UK AHP Support Worker Competency, Education and Career Development Framework (Health Education England (HEE), 2021) are two recent examples of frameworks which aim to address role development and career pathways for AHP CSWs, although they are understandably taking time to embed within workforce practices (Etty et al., 2024).

Whilst there are some published studies evaluating the role of the CSW (Ellis and Connell, 2001; Sarigiovannis et al., 2022) there has not been anything documented on the shared responsibility of student supervision and education. A systematic review by Lizarondo et al. (2010) highlighted CSW responsibilities fall into two main categories: clinical and non-clinical, although supporting students and learners was not included in either of these categories. Similarly, in the recent scoping review by Etty et al. (2024) there was no explicit reference to the CSW role in relation to practice education of students. Generic discussion of ‘team models’ of practice education and student supervision are referenced in published literature, although examples which specifically detail support worker involvement are lacking (Beveridge and Pentland, 2020; Chartered Society of Physiotherapy (CSP) and Royal College of Occupational Therapists (RCOT), 2023).

Close working between occupational therapists and physiotherapists has been noted in many areas of professional practice (Locke et al., 2022; Pighills et al., 2015; Zingmark et al., 2020). A recent scoping review highlighted that where CSWs work across more than one profession, working between OT and PT disciplines was the most common type of combined role across AHP professional groups (Etty et al., 2024). Furthermore, similarities in practice-education have been noted across both professions, with predominance of one-to-one supervision models (Deaves et al., 2024) and shared principles, values and skills (CSP and RCOT, 2023). The likelyhood of transdisciplinary working in practice, along with the potential to experience similar models of practice education, underpinned our reasoning to bring OT and PT CSWs together.

Navigating the ‘Practice Educator’ role for registered health professionals is challenging with general statements from regulatory and professional bodies about the level of education and training required to undertake the role (CSP and RCOT, 2023; HCPC, 2017). This becomes harder still for non-registered staff who often work across disciplinary boundaries and experience the wider challenges outlined above with time, training and recognition for specific dimensions and extensions to their role.

In summary, there are increasing demands on global healthcare workforces and at the same time, there are increasing numbers of learners as part of a strategy to meet longer-term workforce needs. CSWs make up a large part of the workforce and are already responsible for many clinical and non-clinical delegated activities. There are wide variations in how CSW roles are deployed internationally, although there are common themes relating to blurring of boundaries, limited education and training and barriers to effective delegation. Anecdotally, we understand CSWs provide vital support to the facilitation of practice-based learning of students and involvement with learners may be part of their delegated responsibilities, although we have been unable to locate evidence that furthers understanding of this important role. The aims of this project were to (1) explore the support worker role in the practice education of students and (2) to evaluate a training programme for support workers from the perspective of those who completed it.

## Background and context

This project involved three UK Higher Education Institutions (HEIs) in the Northeast and North Cumbria region. All HEIs deliver pre-registration OT and PT programmes. Anecdotally, we understood that students often spent time with CSWs as part of practice-based learning, although before this project, all programmes identified limited engagement with CSWs to understand this involvement, or to support them in this role.

Prior to the project, an advisory group was convened consisting of clinicians, NHS practice placement facilitators, CSWs and academics, with members representing OT and PT and representing different provider organisations across the North-East and North Cumbria region. During the first meeting, the group discussed the rationale for, and content of, the programme and discussed potential evaluation methods to further understand the experience of support workers. The second meeting was utilised to share and discuss the proposed content and to consult on specific questions asked during data collection. In consultation with the advisory group, we developed a pragmatic evaluation approach develop findings that would be practically useful. The project was approved by [Northumbria University; Faculty of Health and Wellbeing] Research Ethics Committee (Ref.: 53137). The study has been reported using the ‘SQUIRE 2.0: Revised standards for quality improvement reporting excellence’ (Ogrinc et al., 2016).

## Educational approach

The concept of ‘Communities of practice’ (Lave and Wenger, 1991) informed our approach to this project. Firstly, we were aware that CSWs were potentially already part of communities of practice in relation to practice education within their own teams and organisations. We hypothesised that CSWs would be acquiring knowledge and skills in supporting students without engagement in formal educational processes, described by Lave and Wenger as *situated cognition.* We therefore began our project by asking CSWs about their current role, experiences and engagement in educational processes.

Lave and Wenger discuss *legitimate peripheral participation* as a way to describe the way in which newer or less experienced members of a community develop mastery and increase their contribution. Although we had anecdotal feedback (from students, practice educators and CSWs themselves) that non-registered staff are members of practice-education communities, we were unable to find evidence in our review of literature, policy and professional standards to support the existence of structures to legitimise this participation and support development in this role.

Therefore, instead of relying solely on the learning that was taking place within existing communities, we wanted to cultivate a new community of practice amongst CSWs from different organisations, with a shared goal of furthering knowledge and skills in practice education. This community would build on existing experiences to share understandings about problems and to create meanings within the group (Gijbels et al., 2022). Importantly, we wanted to create a structure of learning opportunities to provide space for social processes and knowledge sharing, potentially influencing the legitimacy of participation (Lave and Wenger, 1991).

## Methods

### Recruitment to the programme

Information about the programme was circulated through placement or education leads who subsequently approached members of their teams and shared contact details with the academic team delivering the programme. The only essential criteria for participation in the programme were that staff needed to be currently employed in a CSW role where they may have the potential to support OT or PT students. The programme was delivered twice during the academic year 2022–2023, with 24 participants completing the programme from five organisations.

### Programme content

Prior to commencing the programme, participants were emailed a programme information pack including learning outcomes, a programme timetable, contact details for facilitators and links to join online sessions. The programme was scaffolded around an existing NHS e-learning programme for AHP support staff involved in supporting students and therefore the information pack also contained enrolment information for this platform with instructions to enrol on the required module.

**Figure 1.**
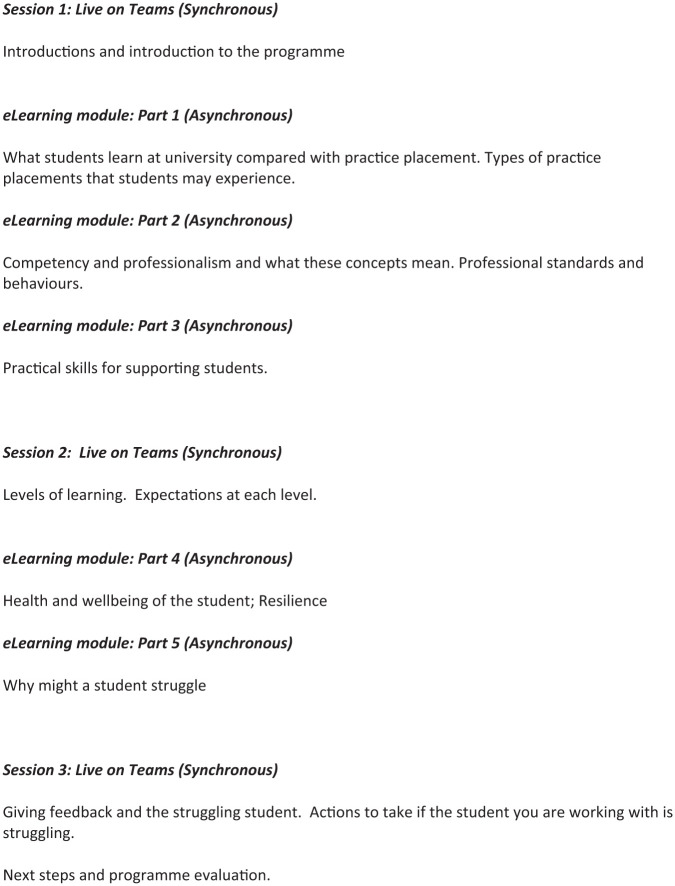
Programme content.

The first online session focussed on participant introductions and an informal sharing of experiences of supporting students. At the end of this session, participants were asked to complete three sections of the e-learning programme focussed on the student journey. The second online session focussed on levels of learning for pre-registration learners, followed by students completing two further sections of e-learning focussed on student health and well-being. The final online session focussed on supporting students with a range of needs on placement, including giving feedback. Each session was facilitated by one member of the project team and was 2 hours in duration. See Figure 1 for an overview of the programme.

### Data collection

Building on consultation with the advisory group, a mixed-method evaluation was developed. Mixed methods research is proposed as appropriate for research which focuses on real-life, contextual understanding and corroborates quantitative data with qualitative explanations (Cresswell and Clark, 2018). Mixed-methods also have utility as an approach to evaluation, with different types of data useful during phases such as needs assessment, programme testing and evaluation of programme impact and processes (Cresswell and Clark, 2018).

The first phase of data collection was through a mixed-method survey, generating both descriptive and exploratory data. Prior to the first session, all participants were sent a participant information sheet by email and a link to the online survey. The first page of the online survey further explained the purpose of the study, how their information would be utilised and asked to verify consent. The aim of the survey was to gather demographic information about the participant group (e.g. role titles, length of time in role), about the nature and extent of involvement in supporting students prior to the training, and to explore perceptions about enablers and challenges when supporting students. The survey consisted of open and closed questions and can be viewed in the Supplemental Material. A first stage of data analysis of survey data was carried out prior to the start of the programme to understand the needs of the group and to flexibly respond to these learning needs within the programme itself.

Prior to the last online session of the programme, participants were resent the same participant information sheet alongside a written consent form and invited to attend an online focus group after the last online session. The aim of the focus group was to explore participants’ perceptions about their role in practice education after undertaking the training, alongside evaluating the content of the training programme in line with their development needs. Focus groups took place on a secure online conferencing platform and audio recordings were transcribed verbatim. This process was followed twice for two cohorts of participants.

### Data analysis

**Figure 2.**
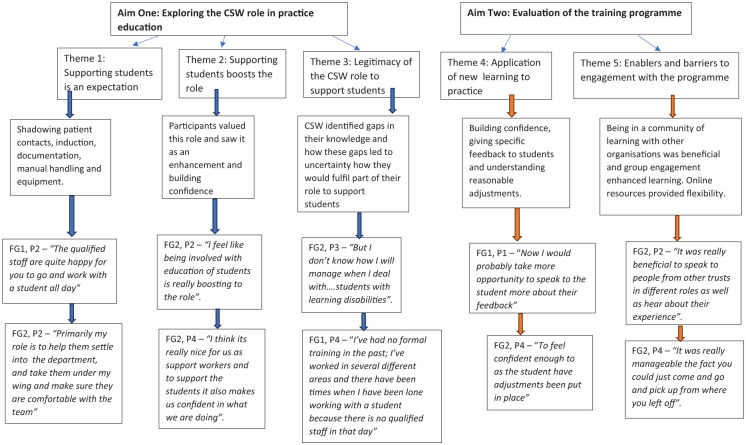
Map of themes and subthemes.

One member of the research team (S.S.) analysed survey data with basic descriptive counting of responses to closed questions and initial coding of responses to open questions. Two further analysts (G.B. and N.B.) carried out independent initial coding of one interview transcript each. Using principles of Thematic Analysis (Braun and Clarke, 2021), all analysts were then involved in reviewing all initial codes from the survey and focus group data. Data analysis meetings were utilised to collapse multiple codes into overarching themes, to re-review all data in line with the themes and to finally define and name themes in line with the research objectives. Quantitative and qualitative data were integrated into five themes. The first three themes relate to the first research objective of exploring the support worker role in supporting practice education of students, with the final two themes relating to the evaluation of the programme. The themes are: *supporting students is an expectation; supporting students ‘boosts the role’; legitimacy of the CSW role to support students; application of new learning to practice;* and *enablers and barriers to engagement with the programme.* A map of themes and subthemes is provided in Figure 2.

## Findings

Seventeen participants completed the online survey, seven participants contributed to the focus group at the end of cohort one and four participants at the end of cohort two. From the 17 survey respondents, 13 participants were in PT assistant or support roles, 3 in OT support roles and 1 participant identified their role as generic. Five respondents had worked in their current role for less than 1 year, 6 had been in the role between 1 and 3 years, 5 between 4 and 6 years and one person had been in the post more than 6 years.

### Exploring the support worker role in the practice education of students

#### Supporting students is an expectation

From the survey, 16 respondents identified that they had previous involvement in supporting students, with only one respondent indicating no experience in this area. When asked to give examples of their involvement, survey responses highlighted shadowing of direct patient contacts, induction and orientation activities to wards and clinical areas, supporting students with tasks such as documentation, and demonstrating specific techniques such as manual handling or using equipment. One response specifically highlighted seeing patients jointly with students who are failing, as they would be unable to work alone.

Involvement in induction and orientation activities was also highlighted in focus group discussions:

Primarily [my role] is to initially is to help them settle into the department and kind of take them under my wing and make sure that they’re happy and comfortable in the team. I think that’s one of the main things before showing them around the wards and things like that. (FG2, P2)

The extract above suggests that support worker involvement during the induction phase has an important pastoral element, with this participant reflecting on their contribution to making sure the student is ‘happy’ and ‘comfortable’ on placement.

The frequency and intensity with which CSWs contribute to student learning were also mentioned:

We sometimes see [students] doing more than what the educators do. We can be spending quite a lot of time with them. (FG2, P4)

[The qualified staff are] quite happy for you to go and work with a student all day. (FG1, P2)

Not only did responses highlight that involvement with students is a routine and regular part of the CSW role, but participants suggested that it was an expected part of their role, and a part that was not always acknowledged.

as an assistant or as a technician or as an associate practitioner, you don’t always get . . . it’s not always acknowledged what you actually do. I think sometimes it’s . . . I’m not speaking for everyone here . . . but sometimes in my experience, it’s ‘the assistant will do it’. (FG1, P2)

#### Supporting students ‘boosts the role’

Despite the reflections noted in the first theme that supporting students was an under-acknowledged part of the support worker role, responses also suggested that participants valued this role and saw it as a valuable role enhancement:

I feel like (being involved with education of students) is really boosting the role of the support worker. (FG2, P2)

The word ‘boost’ here is suggestive of something that not only builds their own confidence, but potentially raises the profile of the role to others and provides an uplift of skills within teams. The link to how working with students contributes to personal confidence, and the perceived value of others having confidence in them, is also clear in the extract below:

I think it’s really nice for us as support workers and to support the students just because it it also makes us confident in what we’re doing. So I think it reassures us that the educator . . . the student educator’s got confidence and trust in us. (FG2, P4)

#### Legitimacy of the CSW role to support students

During focus groups, participants shared examples of gaps in their knowledge and how these gaps led to uncertainty about how they would fulfil parts of the role:

But I don’t know how I will manage when I have to deal with . . . students with learning disabilities (FG2, P3)

So actually to try and plan the day, I’m unsure what they needed at each year and level and what they were working towards. (FG1, P4)

One participant discussed that in the event of giving feedback, they would give this to the educator rather than the student, which again was suggestive of not feeling they had a legitimate voice in these situations:

Prior to this course if I had any feedback on a student – positive or negative – it would just be the educator I would go and speak to. (FG1, P1)

Addressing development needs through training and education was potentially linked to creating a legitimate role:

[I’ve had] no formal training in the past, I’ve worked in several different areas and there have been times when I’ve been lone working with a student because there’s no qualified in that day . . . but now this [training] gives me some sort of ideas so if I ever am in that situation again. (FG1, P4)

Yet survey responses highlighted that only 3 out of 17 respondents had received training in supporting students, and all 3 respondents indicated that this training was of an informal nature.

One participant contrasted the training opportunities available to qualified staff, which legitimise their role as a practice educator. They went on to reflect on how similar opportunities for CSWs contribute to improved practice education experiences for all involved:

As a qualified you have, you can go on the clinical educators course but unfortunately we don’t have that option . . . we’ve now had some training around it which we can use to better that experience for us and them. (FG1, P2)

Another focus group participant gave another example of potentially legitimising their role in working with students linked to their own personal objectives and to appraisal:

It was mentioned as part of my appraisal in terms of developing leadership skills . . . When it comes to like the halfway point [being part of mid-way review meetings] is going to be part of my role moving forward. (FG2, P2)

### Evaluation of the training programme

#### Application of new learning to practice

Within the focus group discussion, participants shared examples of how they would use learning from the course in their involvement with students, with one repeated area relating to how they would use learning from the course to help give feedback:

Prior to this course if I had any feedback on a student – positive or negative – it would just be the educator I would go and speak to about if I’d been working closely with them. But now I’d probably take more opportunity to speak to the student more about their feedback. And to discuss their learning needs and whether they want to take a lead the next day . . . a bit more forthcoming from that point of view I think. (FG1, P1)

Another participant described how learning in the programme had developed awareness of the importance of specificity within feedback:

Say if I was teaching them to put on splints . . . I might say ‘yeah, they were a bit clumsy but the more they practice, the more they’re going to get it’. Or it’s a case of ‘no, they were disinterested, they were looking out of the window’. And I would leave it up to the educator to speak to them about that. Where now, I feel like I’ve got the confidence to be able to break it down into the little steps and to say ‘today I observed you looking around the school, you weren’t concentrating on the child in front of you . . . how do you think that made that child feel?’. (FG1, P3)

Another important area of learning from the course was how knowledge of learning needs and reasonable adjustments would support them when working alongside students:

Now that we know about all these reasonable adjustments that should be made, you know we are support workers . . . [and] should feel confident enough to ask the student have those things been put in place for you?. (FG2, P4)

The suggestion from this participant that they will ask students whether adjustments have been put in place reflects a role in advocating for students and links to an earlier response about their pastoral role in making sure students are happy and comfortable on placement.

Interestingly, application to practice was not only linked to how participants would use learning in relation to their involvement with students. Another example discussed how some of the concepts covered had broader application in relation to teamwork:

And the social styles . . . how to break down social styles . . . and how to communicate in a better way to different social types . . . And I’ll use that now . . . even as part of our team that I’m in now, nevermind the students. (FG1, P3)

#### Enablers and barriers to engagement with the programme

During focus group discussions, participants shared examples of positive elements of the programme which acted as enablers to engagement, alongside elements which they found challenging. One participant shared the reflection that learning together with people from other organisations was a beneficial part of their learning:

I think it was really beneficial to speak to people from other trusts and hospitals in different roles as well to hear about their experiences. So I like that aspect of it. (FG2, P2)

In contrast to this, limited opportunities for interaction were given as a negative of the programme and a potential barrier to positive engagement:

I think it was possibly the first one, where it was very much talk . . . in the chalk and talk thing and I think it was a bit of overload, I agree. I think wherever possible, and I know it’s difficult because you’ve got a time frame, but wherever possible things can be as interactive as possible. (FG1, P6)

The asynchronous value of e-learning as something that could be completed at a convenient time was highlighted as an enabler:

I think it was really manageable and you know the fact that you could just come and go and just pick up where you left off. (FG2, P4)

But whilst there were positive features of the e-learning programme, some participants voiced technical difficulties which acted a barrier to completion:

The only real inconvenience of the e-learning I’ve found is it’s unreliability . . . haven’t been able to do it because the browser was unable to support the e-learning. There’s one particular section of the e-learning that I just cannot complete. There should be a text box for some answers and the text box just isn’t there. (FG1, P7)

The extent of management support was reflected as having the potential to act as either an enabler or barrier to positive engagement with the programme, with contrasting experiences discussed by different participants:

I think it fits into me personally fits into my life. I mean, I only work on a Sunday, Monday, Tuesday. So my my manager has given me just, you know extra. (FG2, P4)

I booked that in with my seniors and at the time it was all fine. But then when it comes to it, they weren’t . . . its’ not like they were funny but sometimes they can be like ‘oh ok’ and they are aware that I have to do it so I do it. But I think sometimes you can just come up against some issues there. (FG1, P2)

## Discussion

This mixed-methods project presents findings which help to explore the CSW role in the practice education of students, alongside evaluating a programme aimed at developing knowledge and skills to support this role. These findings have utility for organisations who wish to prepare and develop those in non-qualified roles regarding their involvement in student education.

Through survey responses and focus group discussions, participants in this study highlighted that they are regularly involved in the education of students, yet this may be an under-acknowledged part of their role. The regularity with which this happens (with reflections that students can be with CSWs a *lot of time*, or *all day*) and the direct support with key professional tasks linked to patient safety (such as record keeping and use of equipment) all indicate that CSWs are likely to be important role models for students. Learning from role models has a significant impact on professional formation (Park et al., 2010). Yet the limited attention given to preparing CSWs for this important role reflects a wider critique that insufficient emphasis is given to the process of learning from role models in healthcare settings (Horsburgh and Ippolito, 2018).

Whilst delegation to CSWs of activities relating to student education seems to be a frequent occurrence, professional standards relating to delegation highlight the importance of the person who is being delegated to being competent in the identified activities (CSP, 2020; RCOT, 2021). Creating supportive learning environments and teaching practice skills are skills in themselves (Fowler, 2018), yet very few participants had received training or development and many reflected uncertainty about the role. This raises critical questions about competence in this area. There have been recent developments to address competency, education and career development of CSW roles (HEE, 2021), although there remains a lack of explicit address of specific competency and professional development in relation to practice education and supporting learners. Critics also highlight that emerging frameworks are also attempting to span large numbers of different AHP disciplines, which has potentially resulted in generic and reductive principles (Etty et al., 2024).

Although participants reflected that their contribution to student education may be under-acknowledged, they simultaneously expressed the value that this element of their role provides, both to them personally, within teams, and to students themselves. Increasing feelings of value and visibility for non-registered workers through the mentoring of others has also been recognised elsewhere (Davison et al., 2024). Nancarrow and Mackey (2005) highlight that patients and families often develop close relationships with CSWs as they see them frequently and they may be less likely to use complicated professional language. Such themes may also resonate with a pastoral role, which adds value to supporting students. Extracts within the theme of *application of new learning to practice* illustrate CSWs finding their own voice and not needing to go through mediators to provide feedback to students, all of which could potentially further strengthen feelings of value and profile over time.

It is important to consider the CSW role carefully and intentionally in relation to supporting practice education so as not to create ambiguity of boundaries or to exacerbate risks from both underutilisation or overburdening (Etty et al., 2024; Mackey and Nancarrow, 2005). Guidance from some professional bodies is emerging (CSP, 2021), although this currently does not reflect the cross-disciplinary nature of the CSW workforce and would benefit from stronger implementation guidance.

E-learning is well recognised as a learning tool across all aspects of healthcare (Fontaine et al., 2019) and this project integrated synchronous and asynchronous e-learning elements. Opportunities to learn with and from others are an important consideration for e-learning (Holmes and Gardner, 2006) and the role of the facilitator to create an environment for contextualisation and participation is crucial (Regmi and Jones, 2020). An important reflection is familiarity and literacy with e-learning, with technological problems or illiteracy likely to compromise the usefulness of professional development opportunities which rely heavily on this approach (Moule et al., 2010).

We return to the concept of communities of practice to contextualise our findings through this pedagogical lens. At the outset, we envisioned that CSWs are already members of communities of practice within their own organisations in relation to the practice education of students. This was reflected in our findings by the roles and responsibilities CSWs are given and that they learn how to carry out these roles often without formal training or support.

The skills of CSWs in relation to caregiving expertise have the potential to centralise their membership of the community, although there is also a danger that this contribution feels invisible (Davidson et al., 2024). Furthermore, wider knowledge is seemingly withheld (such as knowledge of additional learning needs or levels of learning) and therefore CSWs are potentially kept permanently on the periphery of the community. The lack of a formal infrastructure (such as professional standards, educational programmes or clear statements within job descriptions) means membership of practice education communities does not become clear or legitimate, people do not move to full membership and contributions risk being undervalued.

## Recommendations for practice

Whilst recognising the small-scale nature of this study, we encourage all stakeholders involved in the education of health professionals – professional bodies; HEIs; placement provider organisations; individual registered and non-registered staff – to reflect on findings and to work towards practice education communities of practice which are inclusive of CSWs and legitimise their role.

OT and PT professional bodies should explicitly refer to the contribution of CSWs in standards pertaining to practice education and support the differentiation between registered and non-registered staff roles. Organisations can then utilise such guidance within their own role descriptions, competency frameworks and within appraisals to support CSWs to work at the top of their scope of practice and progress to more advanced roles.

Organisations – both placement provider organisations and HEIs – should look for opportunities to create and maintain communities of practice which are inclusive of CSWs. Ensuring CSWs can access and are invited to practice educator updates is an important and achievable step. An inclusive and collaborative approach to planning individual placements should also be considered – knowledge about issues such as level of learning and reasonable adjustments can be shared (and not withheld), but clear roles and responsibilities can also be negotiated.

## Strengths and limitations

This study is the first to our knowledge that specifically focuses on the CSW role in relation to practice education of students and this unique focus is a particular strength. The involvement of an advisory group with representation from multiple perspectives to assist in the design of both the programme and the research methods was another strength. Furthermore, the cross-discipline collaboration between OT and PT, across provider organisations and across HEIs reflects the collaboration required to support practice education. This also reflects the interdisciplinary collaboration CSWs may need if working in generic or shared roles.

This was a small-scale exploratory study, involving a small number of participants in each phase of data collection, meaning transferability beyond this local context may be limited. Also, we did not collect detailed demographic data about participants, which may have enabled a more nuanced and tailored discussion of the development needs for different groups (e.g. whether perspectives and development needs differ according to length of service in role or between CSWs aligned with different disciplines). In addition, focus groups took place within a short time frame after completion of the programme and we therefore do not understand any impact on knowledge and skill development over time. Finally, and in relation to communities of practice, we did not have resources to support this learning community after the end of the project.

Future research could focus on further understanding the contribution of CSWs to practice education, including how the role is planned and delegated. Larger, more objective evaluations are also needed to evaluate the impact of CSW involvement on individual and team workload, capacity to support students and on student performance and experience. We did not ask specific questions about risks or adverse experiences but recognise that the themes of blurred boundaries and unclear delegation mean explicit exploration of this in future research is warranted. Comparative studies that examine optimum ways to support learning and development for CSWs and the role of HEIs and placement provider organisations would help work towards implementation. And studies which evaluate the impact of workforce interventions on other aspects of role development and how learning is sustained over time are also important.

## Conclusion

The findings of this small-scale mixed-methods study suggest that supporting students is a required, but perhaps implicit, part of the CSW role. Despite limited explicit acknowledgement, supporting students is valued by CSWs, supporting them to make a positive contribution to teams and as a vehicle to build confidence and competence in their own abilities. Making this contribution explicit – through formal training, but also by reflecting this in competency frameworks and in role descriptions – could further emphasise the value of this involvement.

A collaborative and tailored programme was evaluated positively by participants, who were able to recognise how this learning could be applied in practice. Participants also identified pragmatic benefits of engaging with a training programme through e-learning and online synchronous sessions. And although such training could be delivered by generic training providers or within trusts or organisations, the opportunity to bring together CSWs from different organisations, and representing diverse models of practice-based learning, is likely to have added and perhaps unseen benefits in addition to those linked to simply learning new knowledge.

**Key findings**

- CSWs in our study are already part of practice education communities of practice, highlighted by the regular responsibilities delegated to them relating to student learning.
- An absence of structures which could legitimise this role (including training opportunities or knowledge-sharing) may mean their contribution feels under-acknowledged or undervalued.
- CSWs do reflect positively on their involvement with students, helping to raise their profile within teams.
- CSWs who undertook a development programme highlighted ways to apply new learning when supporting students.
- Organisations can work towards practice education communities of practice, which are inclusive of CSWs.

**What the study has added**

This study has developed an understanding of how CSWs perceive their role and value in relation to supporting students. Education and training for this role are limited for CSWs, therefore, insights after participating in an education programme highlight ways to address role development and build capacity for practice-based learning within teams.

## Supplemental Material

sj-docx-1-bjo-10.1177_03080226251379996 – Supplemental material for Developing occupational therapy and physiotherapy clinical support workers in their role in supporting student education Supplemental material, sj-docx-1-bjo-10.1177_03080226251379996 for Developing occupational therapy and physiotherapy clinical support workers in their role in supporting student education by Gemma Bradley, Nina Bedding and Sarah Smith in British Journal of Occupational Therapy
