# Supplementary material for: Developing occupational therapy and physiotherapy clinical support workers in their role in supporting student education
Source: Br J Occup Ther. 2025 Oct 27;89(4):264–74. doi: 10.1177/03080226251379996 (PMC12982838; doi:10.1177/03080226251379996)
Supplement: sj-docx-1-bjo-10.1177_03080226251379996 – Supplemental material for Developing occupational therapy and physiotherapy clinical support workers in their role in supporting student education [file sj-docx-1-bjo-10.1177_03080226251379996.docx]

**Developing Allied Health Professional Clinical Support Workers In Their Role In Supporting Students**

**Supplementary File - Online Survey Questions (completed prior to undertaking programme)**

**Questions about current post**

What is your current post?

How long have you been in post?

What profession are you in?

What area of practice do you work in?

**Questions about previous involvement in supporting students**

Have you been involved in supporting students in the past?

Have you received any formal or informal training?

Can you give some examples of your past involvement with students?

Is it formal support or on an ad hoc basis (eg do you know when you will be supporting a student, is it planned)?

Do you support students regularly?

Can you describe the support you receive from your manager/supervisor?

Can you describe what you might be doing with students and how much time you spend with them.

**Questions about perceived enablers and barriers in relation to the role of supporting students**

Is there anything you find difficult when supporting students?

Can you tell us what you would like to be involved in when supporting students?

What would you like to do more of?

What would you like to do less of?

What support do you feel you need to achieve this?
